# High-Sensitivity Low-Cost 2.61 GHz DGS Sensor for Non-Invasive Glucose Level Monitoring †

**DOI:** 10.3390/mi17050543

**Published:** 2026-04-29

**Authors:** Felipe Lucena Souza Medeiros, Alexandre Jean René Serres, Georgina Karla de Freitas Serres, Ravania Luciano Martildes, Caio Vasconcelos Benigno de Abrantes

**Affiliations:** Department of Electrical Engineering, Federal University of Campina Grande, Campina Grande 58429-900, Brazil; felipe.medeiros@ee.ufcg.edu.br (F.L.S.M.); georgina@dee.ufcg.edu.br (G.K.d.F.S.); ravania.martildes@ee.ufcg.edu.br (R.L.M.);

**Keywords:** non-invasive sensor, resonator, microwaves, low cost, diabetes, glucose, glycemic index monitoring

## Abstract

This work presents a loop-shaped (hairpin) resonator incorporating a defective ground structure (DGS) to enhance sensitivity for monitoring water–glucose solutions. The proposed sensor exhibits two resonant frequencies at 2.61 GHz and 4.07 GHz, with reflection coefficients of −46.60 dB and −23.00 dB, respectively. A set of measurements was conducted to compare the performance of the resonator with and without the DGS under two sample-placement configurations: one with water and water–glucose solutions positioned over the feed lines and metallic resonant elements, and another with the water–glucose solutions placed directly over the ground plane. Among the evaluated cases, the ground-plane configuration proved to be the most advantageous, as it produced no frequency shift while yielding distinct magnitude responses of −41.91 dB, −45.62 dB, −47.74 dB, and −49.69 dB for glucose concentrations of 100, 150, 200, and 250 mg/dL, respectively. Overall, the resonator with the defective ground structure consistently demonstrated higher sensitivity and a more stable response pattern, indicating its strong potential for glucose-level monitoring applications.

## 1. Introduction

The evolution of the telecommunications market has been marked by the robustness, efficiency, speed, and reliability of new systems [1], thereby enabling the expansion of applications previously limited to communication and fostering interdisciplinarity, with advances in the fields of medicine [2], energy [3], and automation [4]. In this context, studies seeking to associate the treatment and monitoring of chronic diseases with radiofrequency structures have been widely explored over the past decade, as this monitoring approach enables non-invasive and continuous sensing [5].

Diabetes mellitus is a chronic disease characterized by a metabolic disorder in which the body’s ability to regulate blood glucose is impaired. It can be classified into two main types: type 1, an autoimmune disease in which the pancreas produces little or no insulin [6], and type 2, the most common form of diabetes, which is generally associated with genetic factors, lifestyle, and diet, and is characterized by the body’s resistance to insulin [6].

Insulin is the hormone responsible for transporting glucose into the cells; therefore, a failure in this process leads to an increase in blood glucose concentration [6].

Currently, according to the International Diabetes Federation (IDF), 537 million people aged between 20 and 79 are living with diabetes, and this number is projected to rise to 643 million by 2030 and 783 million by 2045 [7]. This condition can affect the heart, kidneys, blood vessels, eyes, and nerves [6], severely impairing an individual’s quality of life.

**Figure 1:**
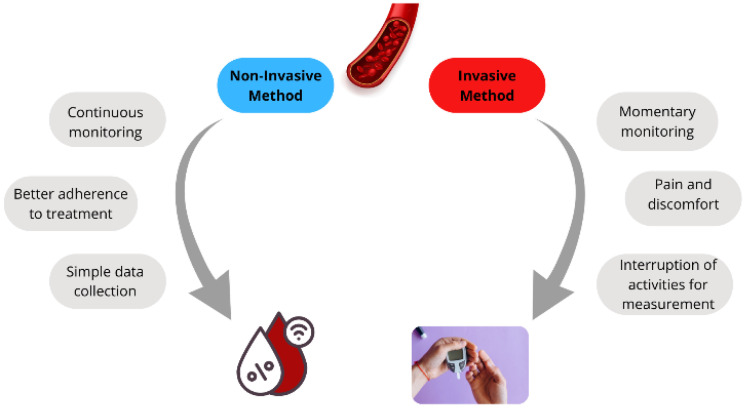
Comparison of invasive and non-invasive methods.

The most common method for glucose monitoring involves pricking the finger with a needle to collect a blood sample, which is then applied to a test strip [5]. Despite the accuracy and widespread acceptance of this invasive method, the discomfort and pain associated with it often lead to resistance, especially among children and adolescents, thereby making treatment adherence more challenging [8]. A comparison between invasive and non-invasive glucose monitoring approaches is shown in Figure 1.

Considering these factors, sensors operating in the microwave frequency range have emerged as a promising alternative for glucose monitoring. These sensors are sensitive to the dielectric properties of materials, such as relative permittivity and equivalent conductivity [9], allowing them to detect and characterize substances such as glucose and its concentration in water or blood [10].

Although recent advances in implantable antennas and miniaturized wireless systems [11] have demonstrated strong potential for continuous in vivo biomedical monitoring, they inherently require surgical intervention. This invasive procedure involves risks such as infection, biocompatibility issues, and physical discomfort. In contrast, the proposed DGS resonator provides a completely non-invasive alternative, enabling robust glucose detection without the need for surgical implantation. This approach is considerably safer and more comfortable, thereby improving patient adherence to continuous monitoring.

Within this context of innovation, this work presents an extended version of the study entitled “Low-Cost DGS Sensor for Non-Invasive Glucose Level Monitoring” [12], now including measurement results. The main objective of that earlier research was to optimize the sensitivity of water–glucose concentration monitoring through the application of a defected ground structure (DGS) in the sensor design. The original study was accepted and published in the 2025 SBMO/IEEE MTT-S International Microwave and Optoelectronics Conference (IMOC) and is available in the IEEE Xplore Digital Library.

Microwave-based technologies offer several advantages for this application, including low-cost generation and detection systems, compatibility with planar technologies, robustness in harsh environments, and the possibility of continuous, non-invasive monitoring. These features are essential for improving patient adherence to treatment and enhancing quality of life.

However, effective glucose monitoring requires highly sensitive structures, since the variations in electrical properties associated with different glucose concentrations are subtle. One way to enhance the sensitivity of such structures is through the implementation of a Defected Ground Structure (DGS). This technique involves intentionally introducing modifications, such as slots or resonant openings, into the ground-plane metallization to improve performance by altering the displacement and distribution of current within the device [10].

Furthermore, when compared with other miniaturization techniques, such as meander lines [13], the Defected Ground Structure (DGS) offers the advantage of modifying the equivalent capacitance and inductance of the circuit without excessively increasing the geometric complexity of the top metallic layer. For sensing applications, this approach helps reduce undesired radiation losses and enhances the concentration of the electric field in the defect region, which is essential for maximizing interaction with the test samples and, consequently, improving sensitivity.

## 2. Materials and Methods

### 2.1. Theory of S-Parameters

Microwave resonators are devices that exhibit enhanced electromagnetic oscillations at specific designed operating frequencies, known as resonance frequencies. They can perform two main functions: generating signals at their resonance frequencies or operating as filters in response to an input signal. The response of these systems is highly sensitive to variations in the properties of materials, water–glucose solutions, and surrounding media in contact with their surfaces, since their behavior under excitation depends on the propagation of the applied electric field.

When resonators operate as filters, they selectively process the frequency components of the input signal by attenuating undesired components located in the stopband while allowing those within the designed passband to propagate. Between these two regions lies the transition band, in which the attenuation varies gradually with frequency.

**Figure 2:**
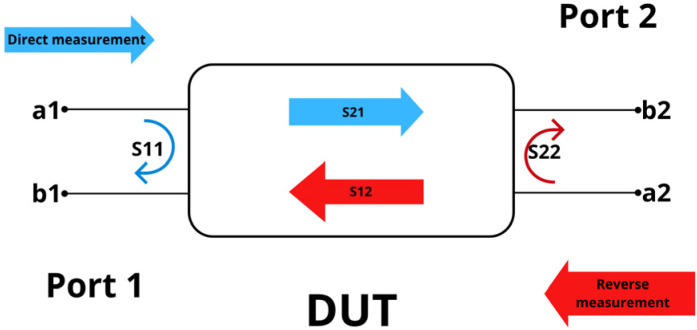
Device Under Test (DUT) Illustration and Two-Port Network Schematic.

In its simplest configuration, a resonator acting as a filter can be represented as a two-port device, with one port corresponding to the signal input and the other to the output. The relationship between the incident waves $a_{x}$ and the reflected waves $b_{x}$ at a given port *x* defines the concept of a DUT (Device under Test) (Figure 2), which serves as the basis for describing the scattering matrix of a two-port network.

Where the S-parameters represent:

$S_{11}:$ input reflection coefficient, or input return loss, at port 1.
$S_{12}$: reverse transmission coefficient, representing the isolation from port 2 to port 1.
$S_{21}$: forward transmission coefficient, representing the transmission from port 1 to port 2.
$S_{22}$: output reflection coefficient, or output return loss, at port 2.

The scattering parameters of the S-matrix can be calculated as follows [14]:

(1) $$S_{11}={\frac{b_{1}}{a_{1}}|}_{a_{2=0}}$$

(2) $$S_{12}={\frac{b_{1}}{a_{2}}|}_{a_{1=0}}$$

(3) $$S_{21}={\frac{b_{2}}{a_{1}}|}_{a_{2=0}}$$

(4) $$S_{22}={\frac{b_{2}}{a_{2}}|}_{a_{1=0}}$$

The amplitudes of the scattering parameters are expressed on a logarithmic scale in decibels (dB) to facilitate visualization, since their magnitude values may vary over several orders of magnitude. The following equation gives the corresponding expression for the amplitude in dB [14].

(5) $$S_{dB}=20{log}_{10}(\left|s\right|)$$

The percentage of transmitted or reflected power is proportional to the square of the corresponding magnitude, as given by the following equations [14].

(6) $$P_{\%}=10^{\frac{S_{dB}}{10}}\times 100$$

Table 1 presents representative values of the scattering parameter $S_{11}$, which is widely used in antenna analysis, together with the corresponding magnitude values and the percentages of reflected and transmitted power.

The table clearly highlights the importance of using a logarithmic scale to present these values, since the percentages of reflected and transmitted power can vary over orders of magnitude.

### 2.2. Geometry of the Sensor

The sensor topology selected in this work is a hairpin resonator, originally developed for filter applications. This choice was motivated by its compact size, narrow bandwidth, and low radiation losses [9].

The initial dimensions of the sensor were analytically determined using standard design equations for microwave hairpin resonators, targeting an operating frequency of 2.45 GHz [9]. These dimensions were then further refined through parametric sweeps in the electromagnetic solver in order to maximize the quality factor and achieve optimal impedance matching.

The narrow bandwidth helps reduce external interference that could affect the circuit response, thereby contributing to greater measurement stability and accuracy for the intended sensing application. In addition, the low radiation losses make it possible to use a low-cost substrate, such as 1.6 mm thick FR4 epoxy, without significantly compromising performance. In this context, improved performance is associated with an increase in the resonance quality factor and peak depth, which ultimately enhances the sensor resolution.

**Figure 3:**
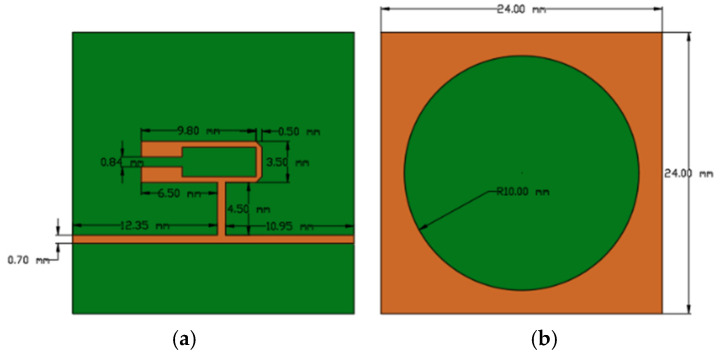
(**a**) Geometry of the hairpin sensor on the top side of the FR4 epoxy substrate, indicating the locations of electrical Ports 1 and 2. (**b**) Bottom side of the substrate showing the ground plane, where the Defected Ground Structure (DGS) is represented by a circular non-metallized region. The orange areas correspond to copper metallization, while the green areas represent the exposed FR4 substrate.

The geometry of the two-port resonator structure, together with the dimensions of the defected ground plane, is shown in Figure 3. The design consists of a square substrate fed by two asymmetric feed lines. A characteristic impedance of 50 Ω was adopted for these feed lines to ensure proper matching with standard microwave measurement equipment.

The proposed structure was modeled in ANSYS^®^ Electronics Desktop 2025 R2 (ANSYS, Inc., Canonsburg, PA, USA), a software platform specialized in high-frequency electromagnetic simulation. The simulation environment was configured using the High-Frequency Structure Simulator (HFSS) with a driven modal solution setup. To minimize artificial reflections, the sensor was enclosed within an air-filled radiation boundary box extending at least λ/4 from the radiating elements. Excitation was applied through 50 Ω lumped ports assigned to the feed lines, and an interpolating frequency sweep from 1 GHz to 6 GHz was used to accurately identify the resonance peaks. In the simulations, the FR4 epoxy substrate was modeled with a relative permittivity ε_r_ = 4.4 and a thickness of 1.6 mm, while the metallization was defined as copper with a thickness of 0.035 mm.

**Figure 4:**
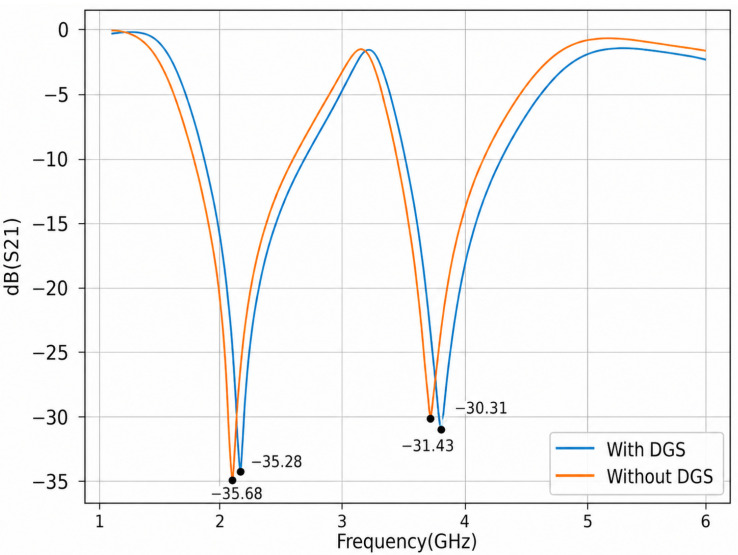
The theoretical frequency dependences of coefficient S_21_, for microwave resonator (DGS vs. without DGS).

As shown in Figure 4, the transmission coefficient response exhibits two pronounced attenuation bands centered at approximately 2.15 GHz and 3.81 GHz. These correspond to the resonance frequencies of the structure, where the signal attenuation reaches −35.82 dB and −31.43 dB, respectively. The introduction of the Defected Ground Structure (DGS) preserves the overall bandwidth characteristics while producing only a slight resonance shift of approximately 0.08 GHz. This indicates that the DGS has a minimal effect on frequency selectivity while improving performance through an increase in the resonance quality factor and peak depth.

### 2.3. Water–Glucose Solution Properties and Sample Configurations

Considering the objective of this work, namely glucose-level monitoring, as well as the challenges posed by diabetes, it is important to consider both normal glucose concentrations, such as 100 mg/dL [15], and more severe hyperglycemic conditions, reaching up to 250 mg/dL [16]. Accordingly, simulations were carried out using water–glucose solutions with the concentrations listed in Table 2, together with their corresponding electrical properties, as reported in [10].

Another important aspect to consider is the ABS plastic container used to hold the water–glucose solutions. The dimensions of this container are presented in Table 3. It is worth noting that ANSYS^®^ Electronics Desktop includes ABS plastic in its material library for simulation purposes. However, the electrical properties of ABS may vary depending on its composition, manufacturer, and supplier. Therefore, it is important to verify the specific type of ABS used in each application. In the present work, the ABS material was modeled in the ANSYS Electronics Desktop environment with a relative permittivity ε_r_ of 2.8 and a dielectric loss tangent (tan δ) of 0.0053.

**Figure 5:**
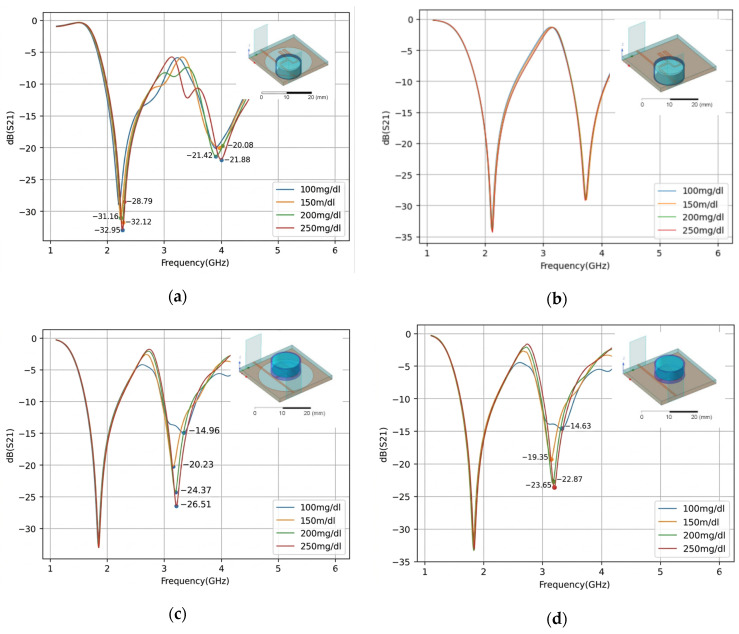
The theoretical frequency dependences of coefficient *S*_21_: (**a**) Structure with DGS and water–glucose solution placed on the ground plane. (**b**) Structure without DGS and water–glucose solution placed on the ground plane. (**c**) Structure with DGS and water–glucose solution placed on top of the structure. (**d**) Structure without DGS and water–glucose solution placed on top of the structure.

Figure 5 illustrates two different configurations for positioning the water–glucose solutions: one in which the solutions are placed on top of the structure, directly above the feed lines and metallic elements, and another in which they are positioned over the ground plane. These configurations also allow a comparative analysis of the sensor sensitivity for structures with and without the inclusion of the Defected Ground Structure (DGS).

The analysis of the obtained results clearly indicates that the structure incorporating the DGS consistently outperformed the configuration without DGS, regardless of the sample positioning. When the water–glucose solutions were placed on top of the structure, directly above the feed lines, a frequency shift was observed. Nevertheless, this configuration still demonstrated excellent sensitivity and a clear differentiation among the glucose concentrations. For the DGS-based structure, the transmission magnitudes recorded at a resonance frequency near 3.2 GHz were −14.96 dB, −20.23 dB, −24.37 dB, and −26.51 dB for glucose concentrations of 100 mg/dL, 150 mg/dL, 200 mg/dL, and 250 mg/dL, respectively. By contrast, the corresponding configuration without DGS yielded magnitudes of −14.63 dB, −19.35 dB, −22.87 dB, and −23.65 dB under the same conditions. This comparison highlights the superior sensitivity of the DGS-based structure, particularly in distinguishing between the two highest concentrations, for which a difference of 2.14 dB was observed, compared with only 0.78 dB for the structure without DGS.

When the water–glucose solutions were positioned directly over the ground plane, only the structure incorporating the DGS exhibited a detectable response, characterized by measurable variations in magnitude near 2.2 GHz. In contrast, the configuration with an intact ground plane produced overlapping response curves with negligible differences, rendering it unsuitable for reliable sensing of glucose concentration levels

### 2.4. Structure Fabrication

The structure was fabricated using the adhesive masking method, in which an adhesive layer is first applied to the metallic regions of the Printed Circuit Board (PCB), followed by immersion of the board in a ferric chloride solution. This process selectively etches the unprotected copper regions, namely the areas not covered by the adhesive mask, leaving only the desired metallic pattern.

**Figure 6:**
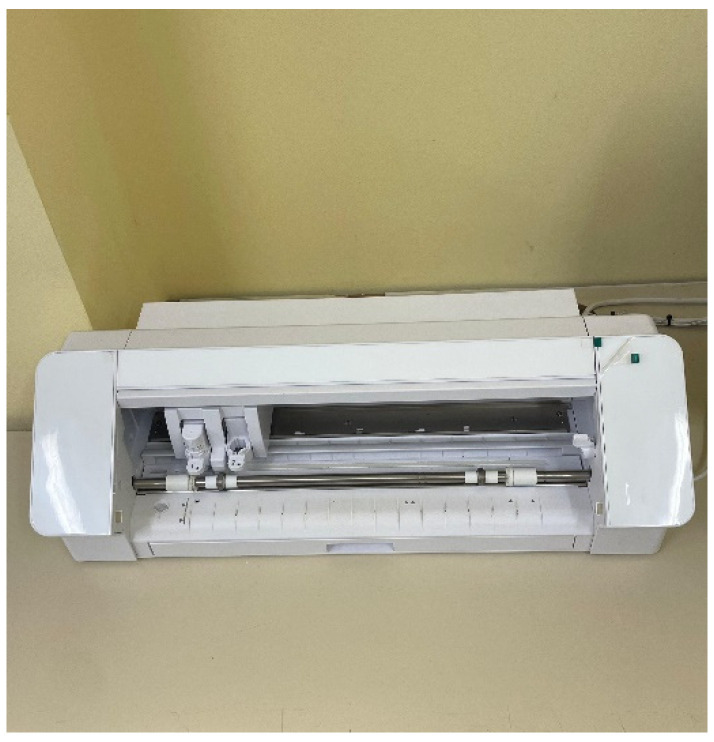
Silhouette Cameo (Silhouette America, Lindon, UT, USA).

The adhesive masks were produced using a Silhouette Cameo cutting plotter (Silhouette America, Lindon, UT, USA) (Figure 6), which is capable of generating customized patterns on materials such as paper, vinyl, fabric, cardstock, acetate, and thin leather. The device is computer-controlled and operated through dedicated software, namely Silhouette Studio (V5.0.081ss Silhouette America, Lindon, UT, USA).

A total of eight resonator structures were fabricated, including four with a DGS and four without DGS. The additional samples were produced because of initial concerns about insufficient adhesion of the masking material to the copper surface, which could have led to defects during the etching process. Nevertheless, all eight fabricated structures preserved their intended geometries, maintained trace integrity, and exhibited identical transverse dimensions.

**Figure 7:**
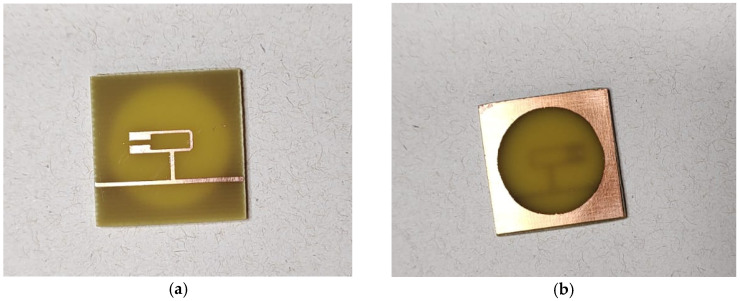
(**a**) Top side of the fabricated resonator, showing the metallic resonant element. (**b**) Bottom side of the fabricated resonator, showing the defected ground plane.

**Figure 8:**
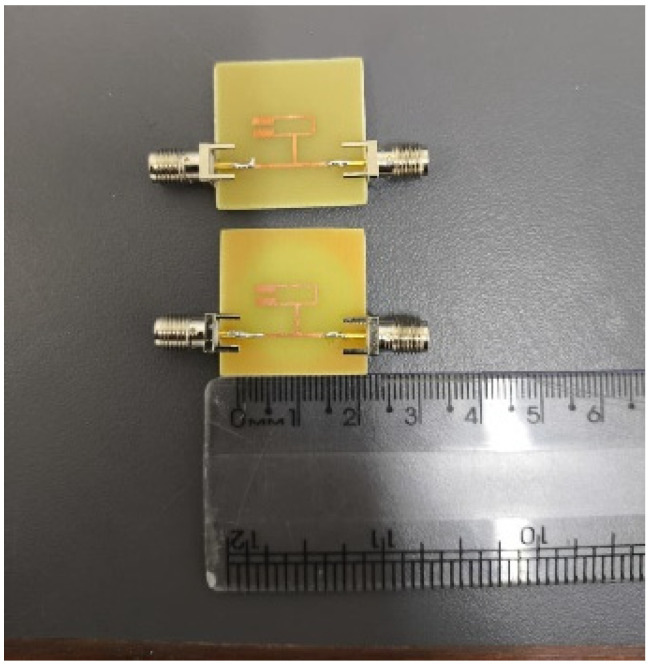
Structures compared using a measurement instrument.

Figure 7a and Figure 7b present, respectively, the top side of the fabricated resonator, showing the metallic resonant element, and the bottom side of the structure, showing the defected ground plane. Figure 8, in turn, provides a comparative illustration of the dimensions of the structures with and without DGS, using a ruler as a reference.

**Figure 9:**
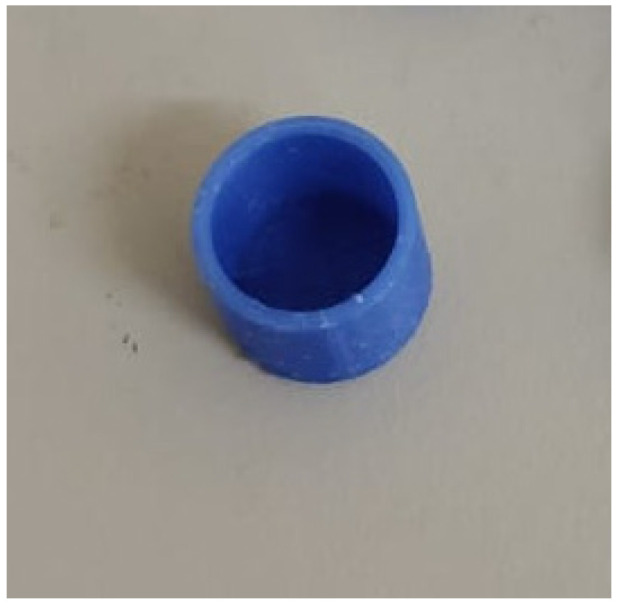
Container manufactured in ABS plastic using a 3D printer.

**Figure 10:**
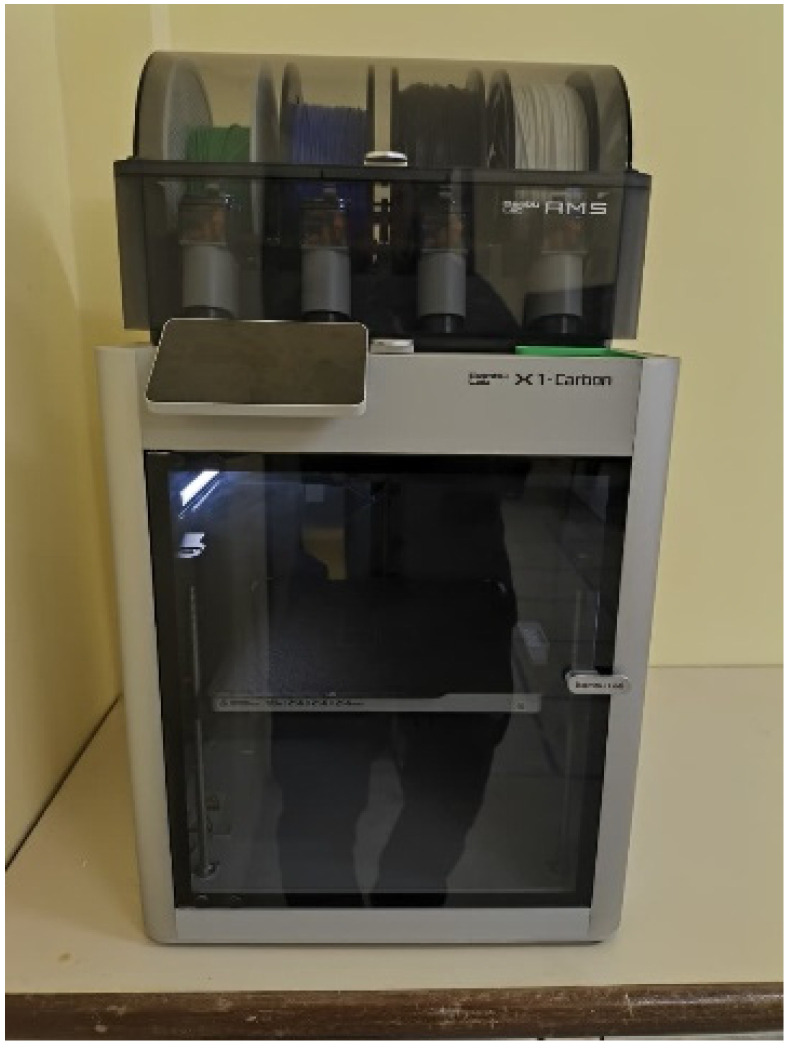
Bambu Lab X1 Carbon printer (Bambu Lab, Shenzhen, China).

Despite their millimetric dimensions and cylindrical geometry, the fabricated containers exhibited good structural strength, as illustrated in Figure 9. The Bambu Lab X1 Carbon printer (Bambu Lab, Shenzhen, China), shown in Figure 10, features an enclosed chamber, making it particularly suitable for printing materials such as ABS with high-dimensional accuracy.

## 3. Results

### Experimental Setup

**Figure 11:**
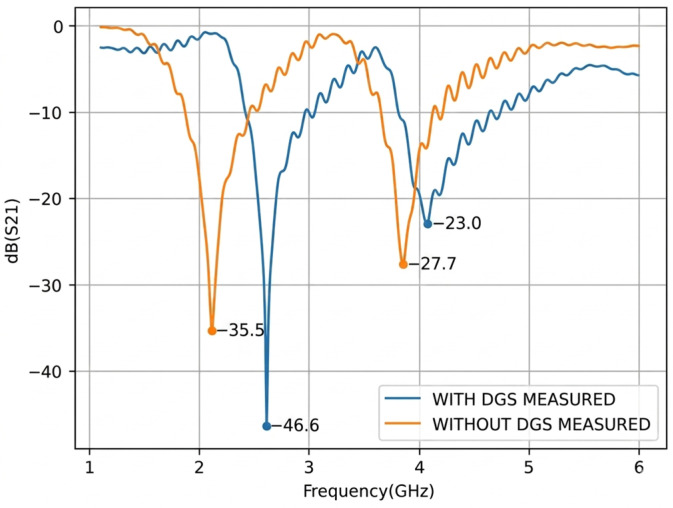
Transmission Parameter *S*_21_ measured with and without DGS.

**Figure 12:**
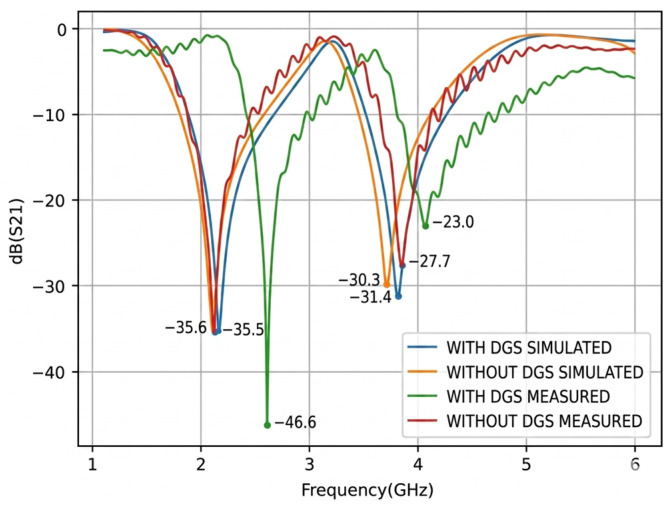
Comparison of Simulated and Measured Transmission Parameter S_21_.

Following the fabrication process, the *S*_21_ parameters were measured using a ZNB40 Vector Network Analyzer (Rohde & Schwarz, München, Germany). Figure 11 presents the measured *S*_21_ responses of the structures with and without the Defected Ground Structure (DGS), while Figure 12 compares the measured results with the previously obtained simulation data.

Upon analysis of the results obtained from the fabricated structures, shown in Figure 12, and their comparison with the simulated results from the previous stage, a good agreement can be observed between the measured and simulated curves for the structures without the Defected Ground Structure (DGS). However, the curve corresponding to the structure with DGS exhibits a frequency shift of approximately 0.45 GHz for the first resonance and 0.26 GHz for the second resonance relative to the simulated response. This discrepancy may be attributed to fabrication tolerances and to variations in the dielectric constant of the FR4 substrate, which may differ from the value assumed in the simulation. In particular, the first measured resonant frequency was observed at 2.61 GHz, whereas the corresponding simulated value was 2.16 GHz.

For the measured structure with DGS, the resonance points occur at 2.61 GHz with a magnitude of −46.60 dB and at 4.07 GHz with a magnitude of −23.00 dB. By contrast, the structure without DGS exhibits two resonances at 2.12 GHz and 3.85 GHz, with magnitudes of −35.50 dB and −27.70 dB, respectively.

During the experimental measurements, precise aliquots of glucose powder were dissolved in distilled water to obtain the desired mass concentrations, ranging from 100 to 250 mg/dL. To ensure concentration accuracy, the water–glucose solutions were weighed using a precision analytical balance. In addition, the volume of liquid dispensed into the container was carefully controlled using a micropipette to maintain a constant liquid column height of approximately 5 mm throughout all measurements.

**Figure 13:**
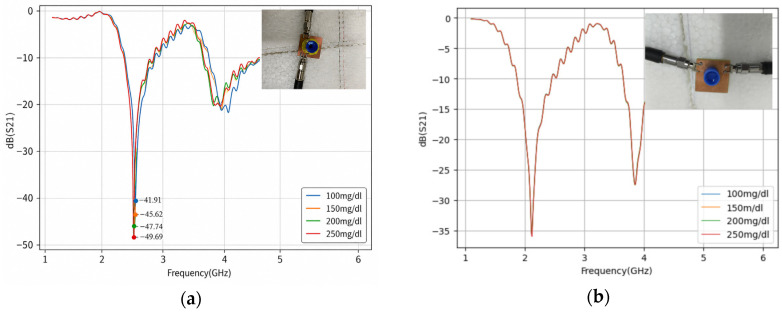

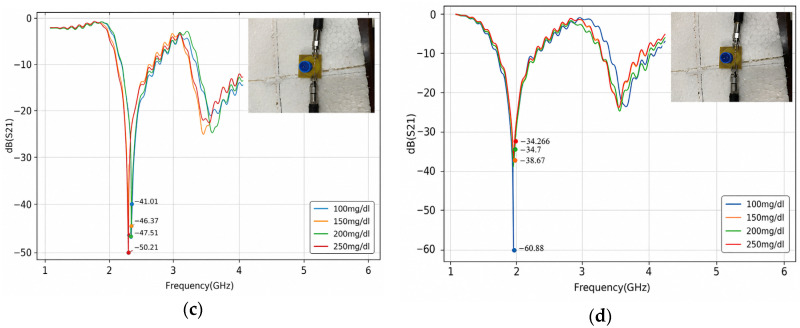
The measured frequency dependences of coefficient S_21_: (**a**) Structure with DGS and water–glucose solution placed on the ground plane. (**b**) Structure without DGS and water–glucose solution placed on the ground plane. (**c**) Structure with DGS and water–glucose solution placed on top of the structure. (**d**) Structure without DGS and water–glucose solution placed on top of the structure.

Figure 13 presents the measured transmission parameter S_21_ for water–glucose solutions with concentrations of 100 mg/dL, 150 mg/dL, 200 mg/dL, and 250 mg/dL, considering each sample-positioning configuration and ground-plane arrangement.

Figure 13 shows the measured results for the structure with and without DGS for two distinct water–glucose solution positioning configurations; the measured results corroborated the initial impressions of the simulated results, showing the structure as more sensitive, stable, and with a clearer pattern; it is important to note that the placement of the cuvette can induce minor shifts in the resonance frequency, highlighting the need for consistent placement.

## 4. Discussion

Upon analysis of the measured results (Figure 13 and Table 4), it is observed that only the first resonance frequency exhibits the capability to sense water–glucose solutions. Furthermore, the worst-performing configuration corresponds to the structure without a DGS, with the water–glucose solution positioned over the ground plane. This observation corroborates the previously simulated results, in which no differentiation between the water–glucose solutions was observed.

The second worst configuration is also associated with the structure without a DGS, but with the water–glucose solution positioned over the metallic element. In this case, the magnitude values for 250 mg/dL and 150 mg/dL are nearly indistinguishable, reaching −34.26 dB and −34.70 dB, respectively. Moreover, the value obtained for the 100 mg/dL water–glucose solution exceeds all the others, reaching −60.88 dB, which indicates a lack of consistent behavior as the solution concentration increases.

The best-performing configurations are associated with structures incorporating a DGS. For both positioning scenarios of the water–glucose solution, a clear trend of increasing magnitude with increasing concentration is observed. Notably, the configuration with the water–glucose solution positioned over the ground plane proves to be particularly advantageous, as it exhibits no frequency shift and yields magnitude values of −41.91 dB, −45.62 dB, −47.74 dB, and −49.69 dB for the 100 mg/dL, 150 mg/dL, 200 mg/dL, and 250 mg/dL water–glucose solutions, respectively. This configuration also exhibits a more consistent response pattern than the one with the water–glucose solution placed on top of the structure, because the DGS ground plane enables stronger interaction with the concentrated electric field in the defected gap, thereby providing higher sensing capability. These results demonstrate a well-defined separation between the magnitude values associated with each water–glucose solution concentration.

The sensitivity ($S_{e}$) of a resonant sensor can be expressed, in simplified form, as:

(7) $$S_{e}=\frac{{∆}_{S21dB}}{{∆}_{mg/dL}}$$

where ${∆}_{S21dB}$ is the variation in amplitude measured at the resonance frequency resulting from a concentration change ${∆}_{mg/dL}$ in water–glucose solution concentration. Structures incorporating a Defected Ground Structure (DGS) generally exhibit higher sensitivity values than conventional configurations, since the increased current path and the stronger concentration of the electric field in the defect region enhance the interaction with the medium under analysis. Among the evaluated cases, the configuration with DGS and the water–glucose solution positioned over the defected ground plane yielded the following sensitivity value:

(8) $$S_{e}=\frac{-49.69-(-41.91)}{250-150}=-0.051\mathrm{dB}/(\mathrm{mg}/\mathrm{dL})$$

Table 5 presents a comparison between the sensitivity of the proposed configuration and those reported in recent studies [17,18,19,20], which investigated different structures and sensing configurations over the past five years. Among the compared approaches, the structure with DGS exhibited the highest sensitivity value, while also demonstrating desirable characteristics for monitoring applications, such as stability and the ability to detect small variations in water–glucose concentration.

As shown in Table 5, the proposed method achieves the highest sensitivity among the compared studies. However, it should be emphasized that our sensor was evaluated over a narrower glucose concentration range (50–250 mg/dL), which directly corresponds to the physiologically relevant range for diabetes monitoring, whereas some of the other reported works consider broader, but less clinically representative, concentration intervals.

When analyzing the values presented in Table 5, it is important to emphasize that the sensitivities are expressed on the decibel scale. In this context, the sensitivity achieved by the structure proposed in this work is particularly noteworthy, since it is able to distinguish responses within the range of approximately −40 dB to −50 dB, thereby demonstrating its capability to detect small variations in concentration, which is a highly desirable characteristic for a precise sensing technology. Another important point is the concentration interval adopted to obtain the reported values, which corresponds to the smallest concentration variations among all the studies compared in the table.

## 5. Conclusions

The obtained results demonstrated that the application of the Defected Ground Structure (DGS) technique to the proposed structure provided a significant gain in sensitivity, enabling precise differentiation among distinct glucose concentrations. In particular, the configuration in which the sample was positioned over the DGS region enabled a more precise differentiation among distinct glucose concentrations, owing to the stronger electromagnetic interaction in that region.

This improvement confirms the effectiveness of the approach and fully meets the objectives established in the work. The simulations conducted provided robust evidence of the solution’s potential for non-invasive glucose monitoring applications. These findings establish a consistent basis for future development of the physical prototype and for conducting experimental tests under real conditions, aiming to validate and expand the practical applicability of the proposed technology.

The measured results allowed confirming the application of DGS as a mechanism capable of enhancing the resonator’s sensing, implying a clear pattern as the water–glucose solutions increase in concentration, meaning an increase in magnitude values for the transmission parameters. Given the obtained results, future work may focus on practical validation under realistic experimental conditions and comparison with conventional invasive methods, in addition to further investigating different DGS geometries, their potential benefits, and their impact on sensor performance.

As a point of future improvement to maximize the device’s performance, replacing the solid-base ABS container with a bottomless plastic cell is highly recommended. By placing this bottomless cell directly on the surface of the non-metallized FR4 substrate, the water–glucose solution will be brought in direct physical contact with the dielectric region of greatest electric field concentration (the DGS gap), eliminating the intermediate ABS layer and potentially increasing the overall sensitivity of the sensor.

## Figures and Tables

**Table 1:** Relationship Between the Scattering Parameter $S_{11}$ in Decibels, Its Magnitude, and the Corresponding Transmitted and Reflected Power Values.

*S*_11_ (dB)	Magnitude	Reflected Power (%)	Transmitted Power (%)
0 dB	1.000	100.0%	0.0%
−3 dB	0.707	50.0%	50.0%
−6 dB	0.501	25.1%	74.9%
−10 dB	0.316	10.0%	90.0%
−15 dB	0.178	3.16%	96.84%
−20 dB	0.100	1.00%	99.00%
−30 dB	0.031	0.10%	99.90%
−40 dB	0.0100	0.01%	99.99%

**Table 2:** The Electrical Properties of Glucose and Water Solutions.

Concentration (mg/dL)	Relative Permittivity (ε_r_)	Conductivity (S/m)	Loss Tangent (tan δ @2.45 GHz)
100	60.67	1.4895	0.157
150	56.29	1.4895	0.156
200	51.92	1.4895	0.155
250	47.54	1.4895	0.154

**Table 3:** ABS Plastic Dimensions.

Dimension	Value (mm)
Inner Diameter	10
Outer Diameter	11
Heigh	5

**Table 4:** Measured Results of the S_21_ Coefficient at the First Resonance Frequency.

Concentration (mg/dL)	Structure with DGS and Water–Glucose Solution Placed on the Ground Plane-S_21_	Structure Without DGS and Water–Glucose Solution Placed on the Ground Plane-S_21_	Structure with DGS and Water–Glucose Solution Placed on Top of the Structure-S_21_	Structure Without DGS and Water–Glucose Solution Placed on Top of the Structure-S_21_
100	−41.91 dB	−36.00 dB	−41.01 dB	−60.88 dB
150	−45.62 dB	−36.00 dB	−46.37 dB	−34.26 dB
200	−47.74 dB	−36.00 dB	−47.51 dB	−34.70 dB
250	−49.69 dB	−36.00 dB	−50.21 dB	−60.88 dB

**Table 5:** Comparison of Sensitivity Values Reported for Microwave-Based Glucose Sensors.

Title	Structure	Frequency	Sensitivity $\left(\left|S_{e}\right|\right)$	Concentration Range
Sensitivity-Enhanced Fluidic Glucose Sensor Based on a Microwave Resonator Coupled with an Interferometric System for Noninvasive and Continuous Detection [17]	Rectangular CSSR	2.26 GHz	0.0194 dB/(mg/dL)	[0–400] mg/dL
PCA-Assisted Blood Glucose Monitoring Using Metamaterial-Inspired Sensor [18]	Metamaterial-Inspired Sensor	3–4 GHz	0.0125 dB/(mg/dL)	[100–300] mg/dL
Noninvasive, Intelligent Blood Glucose Monitoring on Fingertip Using Dual-Band Fusion and LSTM-R Network [19]	ultrawideband (UWB) antennas	3.6 GHz and 8.5 GHz	0.0057 dB/(mg/dL)	[20–500] mg/dL
Combined Approach to Estimate Blood Glucose Level in Noninvasive Monitoring: Ultra-Wide Band Microwave and Cascaded General Regression Neural Network [20]	Ultra-Wideband antenna	0.5–1.37 GHz	0.0039 dB/(mg/dL)	[10–400] mg/dL
High-sensitivity low-cost 2.61 GHz DGS sensor for non-invasive glucose level monitoring. [This work]	Hairpin resonator with defected ground structure	2.61 GHz	0.051 dB/(mg/dL)	[50–250] mg/dL

## Data Availability

The original contributions presented in this study are included in the article. Further inquiries can be directed to the corresponding author.

## Abbreviations

The following abbreviations are used in this manuscript:

DGS	Defected ground structure
DUT	Device under test
PCB	Printed circuit board
IDF	International Diabetes Federation
